# Reference Values of Grip Strength Measured with a Jamar Dynamometer in 1526 Adults with Intellectual Disabilities and Compared to Adults without Intellectual Disability

**DOI:** 10.1371/journal.pone.0129585

**Published:** 2015-06-08

**Authors:** Antonio Cuesta-Vargas, Thessa Hilgenkamp

**Affiliations:** 1 Departamento de Psiquiatría y Fisioterapia, Facultad de Ciencias de la Salud, Universidad de Malaga, Andalucia Tech, Instituto de Biomedicina de Malaga (IBIMA), Grupo de Clinimetria (AE-14), Málaga, Spain; 2 School of Clinical Science, Faculty of Health at the Queensland University of Technology, Brisbane, Australia; 3 Intellectual Disability Medicine, Department of General Practice, Erasmus Medical Center Rotterdam, Rotterdam, The Netherlands; 4 Abrona, Zeist, The Netherlands; SUNY College at Oneonta, UNITED STATES

## Abstract

**Aim:**

The aim of this study was to investigate grip strength in a large sample of people with intellectual disabilities, to establish reference values for adults with intellectual disabilities (ID) and compare it to adults without intellectual disability.

**Methods:**

This study analysed pooled baseline data from two independent studies for all 1526 adults with ID: Special Olympics Funfitness Spain (n = 801) and the Dutch cross-sectional study ‘Healthy aging and intellectual disabilities’ (n = 725).

**Results:**

The grip strength result of people with ID across gender and age subgroups is presented with CI95% values from higher 25.5–31.0 kg in male younger to lower 4.3–21.6 kg in female older.

**Conclusion:**

This study is the first to present grip strength results of a large sample of people with ID from 20–90 years of age. This study provides reference values for people with ID for use in clinical practice.

## Introduction

Intellectual disability is a disability characterised by significant limitations in both intellectual functioning and in adaptive behavior, which covers many everyday social and practical skills. This disability originates before the age of 18, resulting from genetic, neurological, nutritional, social, traumatic or other factors occurring prior to birth, at birth, or during childhood up to the age of brain maturity, that affect intellectual development [1]. High health care costs of this population are caused not only by their lifelong dependence on care and support, but also by higher prevalence rates of a large number of health conditions, compared to the general population [2]. These health conditions are partly related to the cause of the intellectual disability (such as congenital heart defects in Down syndrome, or cerebral palsy), although lifestyle-related health conditions are also highly prevalent [3]. In line with these findings, recent research has shown consistently low levels of physical activity and fitness in people with ID, compared to the general population [4], [5]. These are likely to cause a cascade of health problems and decline of daily functioning, as is described in the conceptual model of Disability-Associated Low Energy Expenditure Deconditioning Syndrome (DALEEDS) [6]. The metabolic effects of physical inactivity, combined with the metabolic effects of antipsychotic drug use, are believed to result in the increased risk of diabetes and other cardiovascular risk factors (metabolic syndrome) in adults with ID [3]. Furthermore, nowadays life expectancy of people with ID is approaching that of the general population, resulting in a larger than ever older population with ID. Their ageing process is generally not accompanied by good health, with higher rates of multimorbidity and frailty at younger ages than the general population. [7]

*Handgrip strength (HGS*), a measure of maximum voluntary force of the hand, has been described as the simplest method in assessing muscle function [8]. It has been shown to be a valid, reliable and feasible measure in multiple populations. It is characterized by overall upper extremity muscle strength [9], and correlates with lower extremity strength and power [10] [11]. Furthermore, this technique has been demonstrated to be a reliable and valid screening tool in the assessment of frailty risk in hospital admission [12] as well as a useful indicator of nutritional status in the non-hospitalised population, particularly in identifying individuals with sarcopenia [13]. It is an important marker in the assessment of sarcopenia [10], nutritional status [14], frailty [15], and muscular strength as a component of physical fitness [16]. Grip strength is a predictor of premature mortality, earlier onset of disability, postoperative complications, increased length of hospital stay [9], fractures, and cognitive decline in older adults [17] [18]. Moreover, data from literature tend to support the fact that HGS may be a good predictor of body cell mass depletion, functional decrease [8] during hospitalization, post-surgery complications, and mortality [19]. Therefore, it might be valuable to introduce this measurement into the population of people with intellectual disability as a marker for sarcopenia, nutritional status, frailty, and physical fitness.

HGS is a non-invasive and quick measurement of grip strength through use of a hand dynamometer, and is increasingly being used in clinical settings, such as in geriatric practice [10]. Measuring grip strength with a hand dynamometer was found to be feasible and reliable in older adults with intellectual disabilities (ID) [20]. More information is required about the reference values on grip strength before introducing grip strength measurement into routine diagnostic work-up of adults with ID. The aims of this study were to investigate grip strength in a large sample of people with intellectual disabilities, to establish reference values for adults with intellectual disabilities (ID) and to compare to adults without intellectual disability across age and gender.

## Methods

### Study design and participants

This study analysed pooled baseline data from two independent studies of people with ID: Special Olympics Funfitness Spain (n = 801) and the Dutch cross-sectional study ‘Healthy aging and intellectual disabilities’ (HA-ID; n = 725). For all 1536 adults with ID, details about design, recruitment and representativeness of the sample have been presented elsewhere [21] [22].

The hours per week of physical activity or sport in Special Olympics Funfitness Spain and physical activity based on pedometer step counts in a subsample of HA-ID were recorded to classify the participants by activity levels.

The waist circumference was measured with a tape measure. Data collection took place between February 2009 and April 2013. Ethical approval was provided by the Medical Ethical Committee of the Erasmus Medical Center (MEC 2008–234) and Ethical Committee of the Faculty of Health Sciences at University of Malaga (FCCSS 314) and by the ethical committees of the participating ID care provider services. Written informed consent was obtained from all participants. This study followed the guidelines of the Declaration of Helsinki (Helsinki, 2008).

### Spanish Special Olympics Funfitness

This study was focused on describing the level of physical fitness of adults with ID and results were compared across gender and level of sports participation.

All of the participants had been diagnosed with mild ID by a specialised doctor and their parent and/or guardian confirmed the diagnosis. All of the individuals appeared to be healthy, which was determined by their health history obtained from the participants and their parent and/or guardian. The exclusion criteria were: 1) any contraindications to exercise as assessed by a medical history questionnaire; 2) documented atherosclerotic heart disease; 3) documented atlantoaxial instability; 4) uncorrected congenital heart disease; and 5) an implanted pacemaker.

Before starting the investigation we guaranteed the protection of confidential information of all participants [Act 15/1999 on Protection of Personal Data]. In all cases we ensured the anonymity of participants. It was also stressed at all times that participation in the study is voluntary and they gave informed consent. All of the participants received counseling and education from the physical therapist and after the screening they were provided with musculoskeletal-specific patient education materials tailored for persons with lower reading levels.

### ‘Healthy aging and intellectual disabilities’ (HA-ID)

This study was conducted to measure a wide range of health aspects of older adults with ID, including level of physical fitness, and results were compared with reference values from the general population.

All of the participants of the HA-ID were 50 years and over, and had been diagnosed with ID (varying from mild to profound). The Revised Physical Activity Readiness Questionnaire (rPARQ) was administered by the professional caregivers in advance of participation in the physical fitness tests, to determine if the participant could participate safely in these tests [23] [24]. Statements about the protection of confidential information and anonymity of participants were included in the informed consent form. It was stressed that participation in this study was voluntary, and that participants could withdraw at any time.

### Procedure

Data were collected as part of an extensive physical fitness assessment. Assessments were guided by test instructors, who all were physiotherapists, occupational therapists or physical activity instructors with experience with individuals with ID. They all received an instruction manual and followed two days of training for the execution of all assessments.

### Handgrip test

Grip strength [25] was measured with the Jamar Hand Dynamometer [26]. Reliability and validity in the general population was good [27] [28]. Test-retest reliability in adults with ID was good (ICC 0.94 [same day interval] and 0.90 [two-week interval]) [20]. In a previous report from the HA-ID study, selective loss of participation was reported. The handle of the dynamometer was placed in the second smallest position according to the instrument’s instructions. The middle phalanges had to rest on the handle, otherwise the position was adjusted. An example of the test was provided by the test instructor squeezing a rubber ball. Subsequently, the participant was allowed to squeeze the ball, to assure understanding of the task. The participant squeezed the dynamometer to his or her maximum ability in seated position, according to the recommendations of the American Society of Hand Therapy ASHT [29]. The best result of three attempts for both the left and right hand (with a one-minute pause between attempts) was recorded, in kilograms (kg). The test instructor had to be convinced that the participant squeezed with maximal effort; otherwise the result was not recorded. In order to check this, test instructors looked at facial expressions, contracting muscles of the arm and hand, the phalanges turning white, and the consistency of the three attempts.

### Comparative reference values from handgrip test for the general population

Two meta-analyses were available for grip strength in the general population, the first provided adult normative values (12 studies, 3317 subjects) [30], the second provided normative values for adults aged 75 years or over (7 studies, 739 subjects) [31]. Means with 95% confidence intervals are presented separately for men and women, for the left hand side and the right hand side, and for 5-year age categories. In these normative values, the distinction between the dominant and the nondominant side is lacking, while the result of the dominant side is widely used to indicate maximum grip strength. To enable comparison with maximum grip strength in our current study, the right hand values of the general population meta-analyses are used as normative values, since this is the dominant side for most people. According to the authors, individual patients whose score is less than the lower limit of the 95% confidence interval of a specific stratum can be considered to be impaired [30], or at least below average [31].

### Statistical analysis

Descriptive statistics including measures of central tendency and dispersion were calculated for the hand grip strength across subgroups. Normal distribution was evaluated with the Kolmogorov–Smirnov test. Analysis was performed with SPSS version 21 for Mac.

## Results

### Descriptives

Participants in this study were 1526 individuals with ID who took part in the HA-ID study and Spanish Special Olympics Games. Characteristics of the study sample are shown in Table 1. The grip strength result for people with ID across gender and age subgroups is presented with CI95% values, and ranges from 25.5–31.0 kg in younger males to 4.3–21.6 kg in older females.

**Table 1:** Descriptive characteristics of study participants (n = 1526).

Descriptive variables	Total sample (n = 1526)	FunFitness sample (n = 801)	HA-ID sample (n = 725)
Gender (n° males, %)	914 (59.5%)	508 (63.5%)	370 (51%)
Age, years (Mean (SD)	46.9 (16.54)	34.3 (9.5)	61.66 (8.01)
Height, meters (Mean (SD)		1.62 (0.12)	1.63 (0.11)
Weight, kilograms) (Mean (SD)		73.1 (16.4)	73.36 (15.7)
Body Mass Index (Mean (SD)		29.2 (5.1)	27.7 (5.3)
Waist Circumference, centimeters (Mean (SD)		95.1 (13.0)	93.5 (15.3)
Physical Activity /hours per week, n° (%)			
Lower 2 hours per week		424 (53.0%)	
Higher 2 hour per week		377 (46.8%)	
HA-ID: Activity based on pedometer step counts			
HA-ID: less than 7500 steps/day			161 (22.2%)
HA-ID: 7500 steps/day or more			90 (12.4%)
HA-ID: unknown			474 (65.4%)

### Reference values

**Fig 1:**
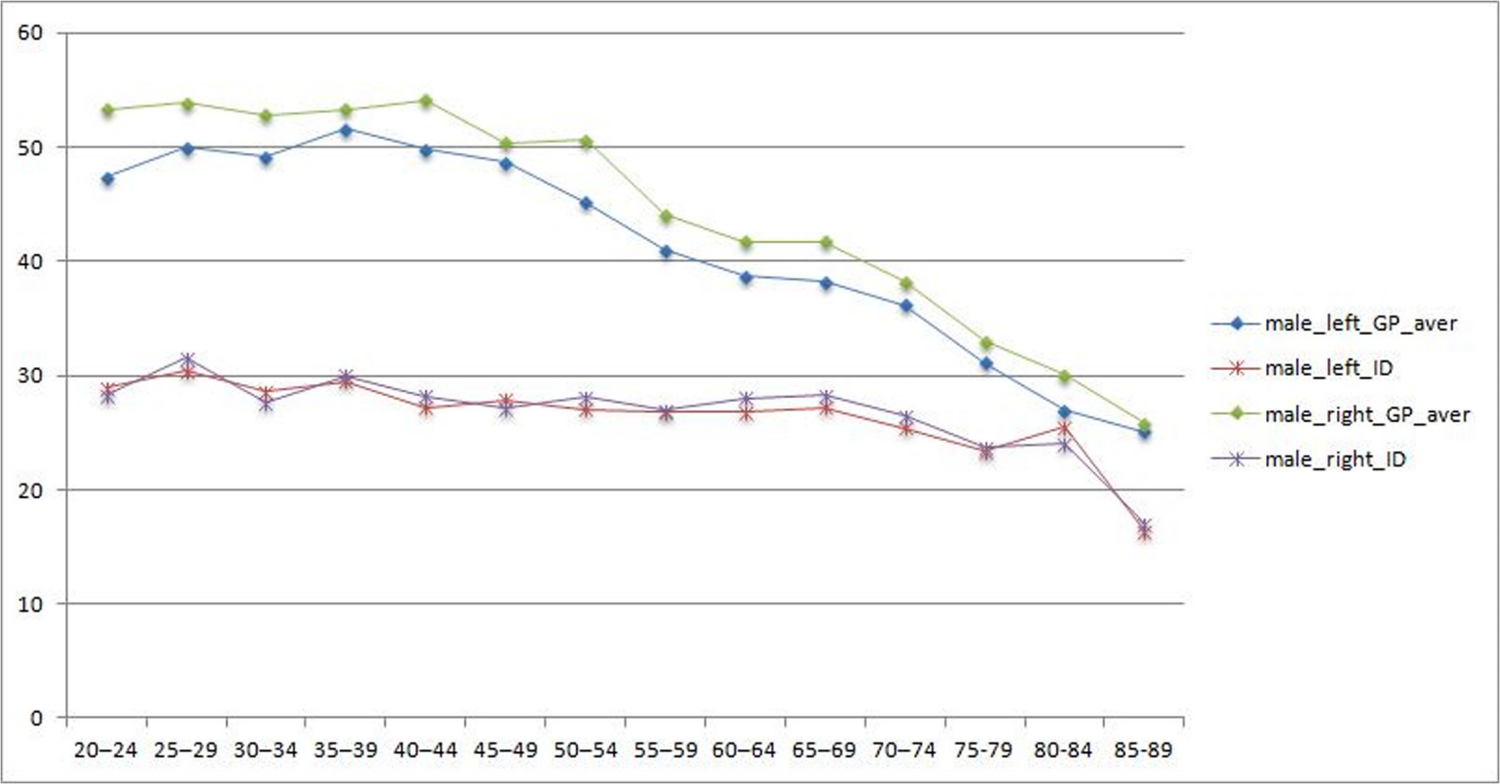
Handgrip values males for adults with ID versus General population.

**Fig 2:**
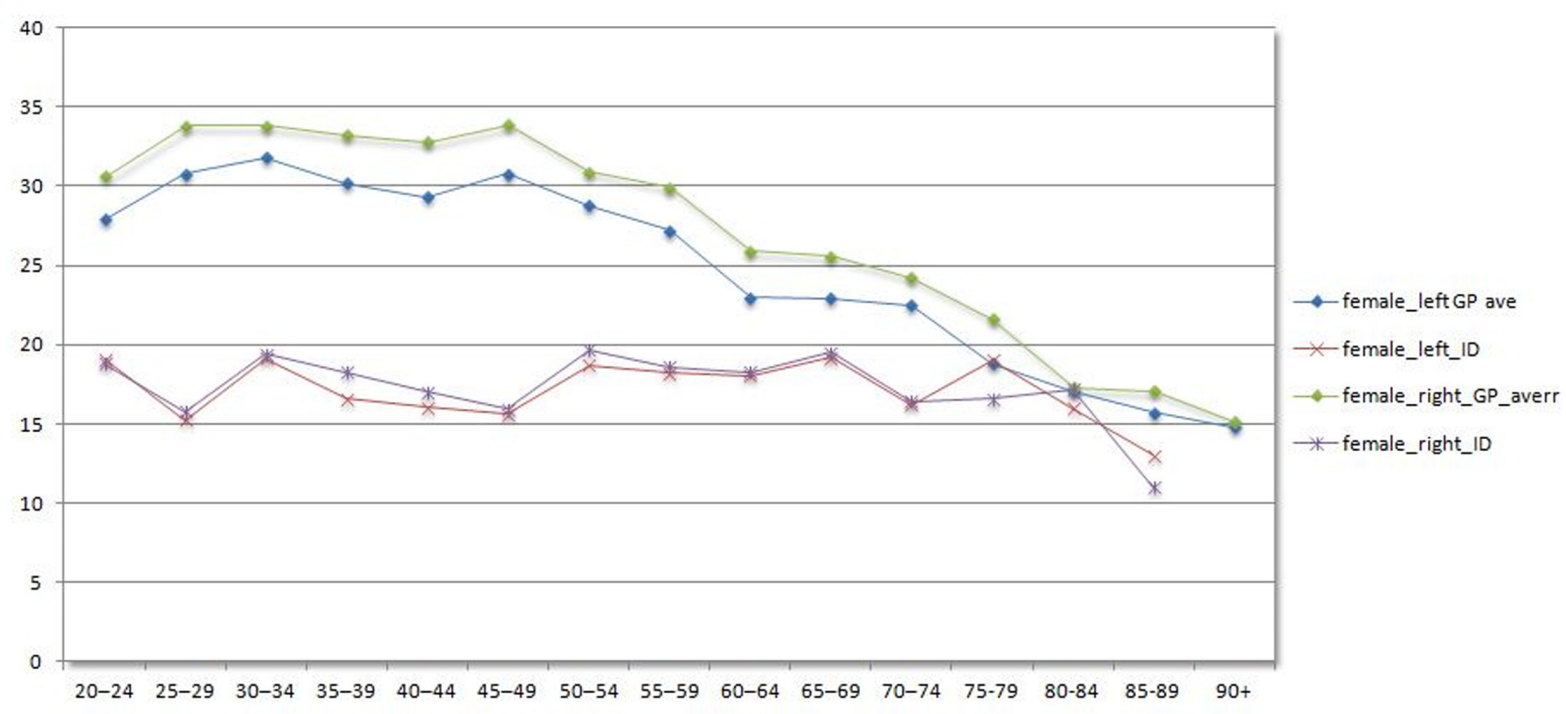
Handgrip values females for adults with ID versus General population.

The grip strength results for people with intellectual disabilities across gender and age categories is presented in Table 2 (males) and Table 3 (females). These results are graphically presented in Fig 1 (males) and Fig 2 (females).

**Table 2:** Mean and CI95% values in kg for females age subgroups in General population (from Bohannon´s studies) and adults with ID.

	General Population (GP)					ID population				
Age	sample size GP	left.GP	95%CI	right.GP	95%CI	Sample size ID (n = 648)	left.ID	95%CI	right.ID	95%CI
20–24	133	27.9	(23.1–32.6)	30.6	(26.7–34.4)	48	19.11	16.8–21.3	18.79	16.4–21.1
25–29	142	30.8	(27.2–34.5)	33.8	(29.5–38.1)	57	15.23	13.0–17.3	15.77	13.8–17.6
30–34	141	31.8	(29.0–34.4)	33.8	(28.9–38.6)	52	19.15	16.9–21.3	19.43	17.4–21.4
35–39	142	30.2	(25.8–34.5)	33.2	(28.6–37.8)	48	16.59	14.0–19.1	18.26	15.2–21.2
40–44	133	29.3	(24.5–34.0)	32.8	(28.0–37.6)	32	16.05	13.1–18.9	17.03	14.0–19.9
45–49	133	30.8	(25.8–35.7)	33.9	(28.9–39.0)	38	15.59	12.6–18.5	15.95	13.4–18.3
50–54	116	28.8	(24.0–33.5)	30.9	(26.7–35.2)	96	18.72	17.0–20.4	19.7	17.9–21.4
55–59	123	27.2	(24.6–29.5)	29.9	(26.4–33.6)	103	18.2	16.7–19.6	18.58	17.0–20.1
60–64	132	23	(18.6–27.3)	25.9	(22.2–29.6)	69	18.07	16.3–19.7	18.28	16.4–20.1
65–69	118	22.9	(19.6–26.2)	25.6	(22.5–28.8)	50	19.19	17.3–20.9	19.52	17.7–21.3
70–74	166	22.5	(19.1–25.8)	24.2	(20.7–27.8)	41	16.22	14.3–18.1	16.41	14.1–18.6
75–79	207	18.8	(14.1–23.5)	21.6	(18.6–24.6)	23	19	16.2–21.7	16.57	12.9–20.2
80–84	166	17.1	(14.5–19.6)	17.3	(14.8–19.9)	5	16	8.4–23.5	17.2	9.3–25.0
85–89	96	15.7	(12.2–19.2)	17.1	(12.8–21.4)	4	13	4.3–21.6	11	0.4–21.5

**Table 3:** Mean and CI95% values in kg for males age subgroups in General population (from Bohannon´s studies) and adults with ID.

	General Population (GP)					ID population				
Age	sample size GP	left.GP	95%CI	right.GP	95%CI	Sample size ID (n = 648)	left.ID	95%CI	right.ID	95%CI
20–24	134	47.4	(38.8–56.1)	53.3	(45.2–61.5)	48	28.89	25.5–31.0	28.3	26.2-31-5
25–29	149	50	(41.1–58.9)	53.9	(44.3–63.6)	57	30.36	28.7–34.3	31.57	27.7–32.9
30–34	120	49.2	(40.4–57.9)	52.8	(44.1–61.5)	52	28.59	25.0–30.2	27.65	26.0–31.1
35–39	117	51.6	(44.0–59.3)	53.3	(44.0–62.6)	48	29.5	27.4–32.4	29.97	27.2–31.7
40–44	111	49.8	(42.5–57.1)	54.1	(47.1–61.2)	32	27.24	25.52–30.8	28.21	24.7–29.6
45–49	110	48.7	(40.3–57.2)	50.4	(42.5–58.3)	38	27.86	23.4–30.7	27.09	24.0–31.6
50–54	100	45.2	(39.4–51.1)	50.6	(44.2–56.9)	96	27.04	25.5–29.0	28.07	24.6–28.9
55–59	100	41	(33.7–48.4)	44.1	(36.7–51.4)	103	26.78	24.9–30.2	27	24.6–28.9
60–64	82	38.7	(33.4–44.0)	41.7	(36.8–46.7)	69	26.78	25.8–29.8	28.01	24.6–29.6
65–69	120	38.2	(32.0–44.4)	41.7	(35.4–47.9)	50	27.17	26.0–27.0	28.28	24.7–29.4
70–74	217	36.2	(30.3–42.1)	38.2	(32.0–44.5)	41	25.43	23.0–29.8	26.44	22.0–28.8
75–79	114	31.1	(25.6–36.6)	33	(27.1–38.9)	23	23.41	20.3–27.0	23.71	20.9–25.0
80–84	107	27	(22.2–31.8)	30.1	(14.8–19.9)	5	25.5	21.3–29.0	24	20.3–28.4
85–89	49	25.1	(20.5–29.7)	25.8	(12.8–21.4)	4	16.33	15.3–29.1	17	15.6–22.1

## Discussion

This study is the first to present grip strength results of a large sample of people with ID from 20–90 years of age. Although this study provides no information on the validity of measuring grip strength for adverse health outcomes in people with ID, it does provide reference values for people with ID for use in clinical practice. In line with the suggestion provided by Bohannon for the general population, scoring below the 95% confidence interval of the appropriate gender and age category reflects a below average result for that individual.

In the comparison with the data for the general population, this study demonstrates that people with intellectual disabilites have very low levels of grip strength during their entire life. Even at an age of 20–30 years, their grip strength is as low as for 75 year-olds of the general population, which is the age of a nursing home population. There is only a slight decline across the ages, not nearly as much as in the general population. This low level of grip strength probably represents the bare minimum of grip strength necessary to perform basic daily activities. It raises the question whether sarcopenia is already happening at 25, or whether people with ID have never built up any muscle mass to begin with due to physical inactivity.As mentioned in the introduction, grip strength is a strong predictor for a number of negative future health outcomes in the general population [10,13]. Research on this topic in people with ID is scarce, and somewhat contradictory, which might be explained by the consistently low levels across the entire life span. Oppewal et al. did not find grip strength to be predictive of a decline in basic activities of daily living [32] or falls [33], but it did prove to be predictive of instrumental activities of daily living [32].

One of the strengths of this study is the large dataset used, resulting from combining two highly comparable samples with regards to study procedures, physical activity level, and data collection.

Limitations of this study are the lack of information on the presence of Down syndrome, which has been demonstrated to negatively influence grip strength [34]. Since the prevalence of Down syndrome is only around 15% of the total population of people with ID, this influence is considered to be minor. The level of intellectual disability does seem to influence grip strength results [34], but the level of ID was not available for full sample in the Fun Fitness ID sample. The second limitation is the comparison with published reference data of the general population. Not being able to work with raw data hampers statistical comparisons. In line with this, the lack of information about physical activity levels of the general population is also a problem, since differences in physical activity levels between the two samples could have confounded the difference in grip strength. A third limitation is reporting the grip strength for the left and the right hand, not taking differences in handedness into account. In the general population, most people are right-handed, and often this is also the strongest hand. In people with intellectual disabilities, not only is a larger percentage left-handed or has no preference for either the right or left hand, but also this does not necessarily result in this hand being the strongest hand [35]. We recommend measuring grip strength in both hands for people with ID, and comparing the results with the reference values of both left and right hand.

Overall, these results provide more insight into the development of grip strength across age in people with ID, compared to the development of grip strength in the general population. The implications of these findings for policy and practice are significant, and underline the importance of constant focus on promoting physical activity and exercise across the entire population with ID.
